# Incidence of childhood leukaemia in the vicinity of nuclear sites in France, 1990–1998

**DOI:** 10.1038/sj.bjc.6602068

**Published:** 2004-07-27

**Authors:** M L White-Koning, D Hémon, D Laurier, M Tirmarche, E Jougla, A Goubin, J Clavel

**Affiliations:** 1 Institut National de la Santé et de la Recherche Médicale INSERM – U170-IFR69, 16 avenue Paul Vaillant Couturier, 94807 Villejuif Cedex, France; 2 Institut de Radioprotection et de Sûreté Nucléaire, IRSN/DRPH/SRBE-LePID, Fontenay aux Roses, France; 3 Institut National de la Santé et de la Recherche Médicale INSERM-CépiDc-IFR69, Le Vésinet, France

**Keywords:** childhood leukaemia, nuclear sites, incidence

## Abstract

Overall, 670 cases (*O*) of childhood leukaemia were diagnosed within 20 km of the 29 French nuclear installations between 1990 and 1998 compared to an expected number (*E*) of 729.09 cases (*O/E*=0.92, 95% confidence interval (CI)=[0.85–0.99]). Each of the four areas defined around the sites showed non significant deficits of cases (0–5 km: *O*=65, *O/E*=0.87, CI=[0.67–1.10]; 5–10 km: *O*=165, *O/E*=0.95, CI=[0.81–1.10]; 10–15 km: *O*=220, *O/E*=0.88, CI=[0.77–1.00]; 15–20 km: *O*=220, *O/E*=0.96, CI=[0.84–1.10]). There was no evidence of a trend in standardised incidence ratio with distance from the sites for all children or for any of the three age groups studied. Similar results were obtained when the start-up year of the electricity-generating nuclear sites and their electric nuclear power were taken into account. No evidence was found of a generally increased risk of childhood leukaemia around the 29 French nuclear sites under study during 1990–1998.

Reports of an increased incidence of leukaemia among young people living near the nuclear site of Sellafield lead to an extensive investigation of this area (COMARE, 1996) and the sites of Dounreay (COMARE, 1988), Aldermaston and Burghfield (COMARE, 1989), 20 years ago. Radiological studies showed that the levels of radioactivity in these areas were far below those necessary to account for the observed excesses (Dionan *et al*, 1986, 1987; Simmonds *et al*, 1995). Kinlen (1988, 1995) and Kinlen *et al* (1995) hypothesised that the high rates of population mixing due to the construction of the sites may induce local epidemics of an unknown infective agent. Although some results are consistent with this hypothesis (Dickinson and Parker, 1999; Boutou *et al*, 2002), the underlying biological mechanism has yet to be determined. Several studies have systematically examined the evidence relating to all of a country's nuclear installations. Mortality studies in the USA (Jablon *et al*, 1991), Canada (McLaughlin *et al*, 1993), France (Hattchouel *et al*, 1995), Spain (Lopez-Abente *et al*, 1999) and Japan (Iwasaki *et al*, 1995), and incidence studies in England and Wales (Bithell *et al*, 1994), Scotland (Sharp *et al*, 1996), Germany (Michaelis *et al*, 1992; Kaatsch *et al*, 1998), Sweden (Waller *et al*, 1995), Canada (McLaughlin *et al*, 1993) and the USA (Jablon *et al*, 1991) found no statistical evidence of an excess of leukaemia among children living around nuclear sites. In France, despite indications of increased incidence for certain combinations of age groups and geographical areas, extensive investigation of the La Hague site (Viel and Richardson, 1990; Viel *et al*, 1993, 1995; Pobel and Viel, 1997; Guizard *et al*, 2001; Boutou *et al*, 2002) finally yielded, as for the Marcoule site (Bouges *et al*, 1999), no evidence of a significant excess of cases of childhood leukaemia. The present paper reports the first systematic study of the incidence of childhood leukaemia around all 29 French nuclear installations.

## MATERIALS AND METHODS

The study was designed to investigate the incidence of leukaemia among children under 15 years of age living less than 20 km away from one of the 29 nuclear installations in France and, in particular, to examine the existence of a trend in standardised incidence ratio (SIR) with increasing distance from the sites.

It included all cases of acute leukaemia diagnosed between the 1st January 1990 and 31st December 1998 among children under 15 years of age living within the study area at diagnosis. They were provided by the National Registry of Childhood Leukaemia and Lymphoma (INSERM U170, J Clavel), which has registered all cases diagnosed in France since 1st January 1990 (Clavel *et al*, 2004).

Table 1

**Table 1:** Distribution according to distance of observed (*O)* and expected (*E*) cases of leukaemia among children aged 0–14 years living less than 20 km away from one of the 29 nuclear sites in France (1990–1998)

	**0–5 km**		**5–10 km**		**10–15 km**		**15–20 km**		**Total**	
**Nuclear sites (year^a^, power^b^)**	***O***	***E***	***O***	***E***	***O***	***E***	***O***	***E***	***O***	***E***
*EGNS:*										
Belleville (1987, 2600)	0	0.26	0	0.53	2	1.44	3	1.05	5	3.28
Bugey (1971, 3600)	0	0.21	2	2.62	5	3.67	7	5.58	14	12.07
Cattenom (1986, 5200)	0	0.92	5	5.95	3	4.25	3	6.48	11	17.61
Chinon (1963, 3600)	1	0.68	6	1.47	2	0.94	5	3.55	14	6.64
Chooz (1966, 2800)	0	0.59	0	0.75	0	0.54	0	0.01	0	1.90
Civaux (1997, 2800)	0	0.08	1	0.51	1	0.88	5	1.48	7	2.95
Cruas (1983, 3600)	0	0.48	3	3.21	4	2.20	3	3.18	10	9.07
Dampierre (1980, 3600)	0	0.38	2	1.48	1	1.02	0	1.07	3	3.95
Fessenheim (1977, 1800)	0	0.36	0	0.68	0	0.88	0	3.59	0	5.51
Flamanville (1985, 2600)	0	0.29	1	0.63	0	0.53	1	1.02	2	2.48
Golfech (1990, 2600)	0	0.53	1	0.57	1	0.87	2	2.37	4	4.33
Gravelines (1980, 5400)	1	1.67	2	2.03	6	6.49	5	10.12	14	20.31
Le Blayais (1981, 3600)	0	0.00	1	1.09	2	1.55	1	1.28	4	3.91
Nogent (1987, 2600)	1	0.55	2	0.60	0	0.77	1	2.98	4	4.90
Paluel (1984, 5200)	0	0.21	1	1.17	0	0.69	2	1.26	3	3.34
Penly (1990, 2600)	0	0.42	1	0.85	3	4.17	5	2.21	9	7.65
St Alban (1985, 2600)	4	1.69	4	2.56	0	1.88	4	9.59	12	15.72
St Laurent (1969, 1800)	1	0.43	0	1.28	2	1.36	2	1.41	5	4.48
Tricastin/Pierrelatte	0	0.88	2	3.17	1	2.09	1	0.79	4	6.92
(1980, 3600)										
										
Total EGNS	8	10.64	34	31.13	33	36.21	50	59.03	125	137.01
SIR (95% CI)	0.75	(0.32–1.48)	1.09	(0.76–1.53)	0.91	(0.63–1.28)	0.85	(0.63–1.12)	0.91	(0.76–1.09)
										
*Other nuclear sites*										
Cadarache (1963)	0	0.05	1	0.70	1	0.86	2	2.49	4	4.09
Creys-Malville (1985)	1	0.19	1	0.94	1	0.66	5	2.41	8	4.20
Grenoble (1956)	14	14.47	9	11.10	5	4.13	10	7.48	38	37.18
La Hague (1967)	2	0.31	0	0.43	1	0.73	2	5.22	5	6.69
Marcoule (1956)	0	0.19	5	4.89	5	2.11	1	1.95	11	9.14
Romans-sur-Isère (1962)	2	3.79	1	0.76	3	2.32	2	2.03	8	8.90
Valduc (1962)	0	0.03	0	0.09	0	0.21	0	0.64	0	0.96
Bruyères/Saclay/Fontenay	38	45.43	114	124.25	171	203.54	148	147.68	471	520.91
(1955/1950/1948)										
										
Subtotal (EGNS and other except B/S/F^c^)	27	29.68	51	50.05	49	47.22	72	81.24	199	208.18
SIR (95% CI)	0.91	(0.60–1.32)	1.02	(0.76–1.34)	1.04	(0.77–1.37)	0.89	(0.69–1.12)	0.96	(0.83–1.10)
										
Total (EGNS and other)	65	75.11	165	174.30	220	250.76	220	228.92	670	729.09
SIR (95% CI)	*0.87*	(0.67–1.10)	0.95	(0.81–1.10)	0.88	(0.77–1.00)	0.96	(0.84–1.10)	0.92	(0.85–0.99)

EGNS=electricity-generating nuclear sites; SIR=standardised incidence ratio; (95% CI)=95% confidence interval for the SIR.

a Year of start-up.

b Electric nuclear power in MWe of an EGNS given by the number of units on site multiplied by the power of each unit.

c B/S/F=Bruyères/Saclay/Fontenay.

lists the 29 French nuclear sites considered in this study. Each of these sites included at least one ‘regulated nuclear facility’-classified reactor in activity for 1 year at least during the study period (1990–1998).

All 19 electricity-generating nuclear sites (EGNS), which are owned and operated by ‘*Electricité de France*’ (EDF), were started up before the beginning of our study period except for Golfech (1990), Penly (1990) and Civaux (1997). In addition to the start-up year, we considered the electric nuclear power (in electric mega Watts (MWe)) of each of these sites, which is the sum of the power of all the units on site (Table 1). Note that the site of Tricastin started producing electricity in 1980, but it also includes plants (such as Pierrelatte) involved in uranium enrichment and conversion as well as other research activities, which started in 1960. The site of Bugey has also been referred to under the name of St Vulbas (Hill and Laplanche, 1990); we shall follow the current (and most common) usage and refer to it as Bugey.

The remaining 10 nuclear installations are of various types: Romans-sur-Isère is a nuclear fuel-processing plant operated by the ‘*Société Franco-Belge de Fabrication de Combustibles*’ (FBFC), Marcoule is a nuclear fuel-processing plant operated by the ‘*Compagnie Générale des Matières Nucléaires*’ (COGEMA) and also includes research activities, La Hague is a nuclear fuel-reprocessing plant operated by COGEMA, Creys-Malville was a fast neutron reactor designed to produce electricity and plutonium operated by EDF, and the six remaining sites (Bruyères-le-Chatel, Cadarache, Fontenay-aux-Roses, Grenoble, Saclay and Valduc) are nuclear research centres operated by the ‘*Commissariat à l'Energie Atomique*’ (CEA).

A pooled analysis of the 29 sites was carried out, followed by an individual study of each site. The 19 EGNS were analysed as a separate subgroup because of their common characteristics. Further analysis was carried out in this subgroup according to the start-up year and electric nuclear power of the sites.

The areas under study were discs of radii 20 km centred on the nuclear sites. These were subsequently divided into concentric bands (0–5, 5–10, 10–15 and 15–20 km). The smallest administrative units for which sex- and age-specific population counts are available in France are the ‘communes’. There are 36565 communes in France with an average population per commune of 1609 inhabitants; 64% of all communes are considered rural (average population of 600). The study areas were constructed as aggregations of the communes whose town hall was within the defined zones. The 5 km bands were chosen because this accorded with previous studies and without prior knowledge of the geographical distribution of cases of leukaemia.

When the study areas around two sites overlapped (this occurred six times), the communes were assigned to the nearest of the two sites in order to maintain a strict partition of the areas under study, thus ensuring independence of the statistical tests. The nuclear research sites of Bruyères-le-Châtel (B), Saclay (S) and Fontenay (F) were treated differently as all three are within close distance of each other (distance between B and F: 21.6 km; B–S: 14.6 km; S–F: 11.3 km). As the three study areas (discs of radii 20 km) had considerable overlaps, we decided to consider these sites collectively. The 0–5 km zone contained the communes whose distance to the closest of the three sites was less than 5 km, the same rule applying for each of the study zones. Throughout the study these three nuclear plants were considered as one site, which explains the reference to 27 sites rather than the original 29. It should also be noted that the sites of Chooz and Fessenheim are close, respectively, to the Belgian and to the German borders, but only the French part of the area was taken into account.

For each commune, age- and sex-specific population counts were obtained from the French National Institute of Economic and Statistical Studies (INSEE) for the March 1990 and March 1999 censuses, as well as the number of births according to sex (INSEE) and the number of deaths according to sex and age (CépiDc, INSERM) for each year from 1990 to 1999. A diagonal interpolation procedure (Benhamou and Laplanche, 1991) was developed in order to obtain age-specific population estimates for years 1991–1998 for each commune. Each of the individual age cohorts was followed up from the 1990 census or from birth by ageing 1 year at a time and subtracting the number of deaths, which occurred during that year in the given age cohort. We estimated a migration factor in order to construct the final age-specific population estimates. The population at risk for a given year and a given commune were subsequently calculated using these estimates. National age-specific incidence rates based on the National Registry data were used to derive annual expected numbers of cases for each age group and commune under study.

The relative risk of leukaemia was estimated by the SIR, defined as the ratio of observed (*O*) over expected (*E*) number of cases. The 95% confidence intervals (CI) for these ratios were given using Byar's approximation (Breslow and Day, 1987).

Our principal aim was to investigate the existence of a decrease in the SIR of childhood leukaemia with increasing distance from the nuclear installations. This was carried out using three tests as follows: the likelihood ratio test, a Poisson regression test using inverse distance and Stone's Poisson maximum test. The likelihood ratio test based on the Poisson log-linear regression models test was used to examine the heterogeneity between predefined areas around the sites. The second test, also based on the Poisson regression, uses inverse distance as a surrogate for exposure. This test belongs to the class of linear risk score tests defined by Bithell (1995). Stone's Poisson maximum test follows a nonparametric approach and is based on the maximum value of the SIR as one aggregates areas ordered by distance from the site into a region of increasing size (Stone, 1988). The latter two tests differ from the likelihood ratio test in that they explicitly test for a decrease in SIR rather than just testing for heterogeneity. These two tests were applied to the predefined four area classification around the sites and also to concentric bands of width 1 km.

For all three tests, we used both an external and an internal reference, alternatively called unconditional and conditional forms of a test (Morris and Wakefield, 2000). Conditional tests correct for the local level of risk, thus ignoring the extent to which the overall observed number of cases around a given site differs from the overall expected number, and consider only the distribution of cases within the study region. On the other hand, unconditional tests are sensitive both to any excess risk in the overall study region compared to the external reference and to the spatial pattern of observed cases.

The analysis of childhood leukaemia incidence around all French nuclear installations was our main objective. However, the 29 sites under study are of different types and there is a strong effect of age on childhood leukaemia. This probable heterogeneity called for a more detailed study of childhood leukaemia incidence around the sites. Analyses were thus carried out according to the type of site and according to three age groups (0–4, 5–9 and 10–14 years). For EGNS, additional analyses were performed in order to consider potential variations according to the electrical power (1800, 2600–2800, 3600, 5200–5400 MWe) and the period of start-up (before 1980, 1980–1984, 1985–1989, 1990 and later). Bonferroni's method was used in order to correct for multiple testing.

The statistical power of the study for finding an excess of cases and a decrease in SIR with increasing distance was examined according to two types of alternative hypotheses using simulation methods (see Appendix A).

## RESULTS

Table 1 shows the distribution of observed and expected cases of leukaemia among children under 15 years of age around the 29 French nuclear sites. Altogether, 670 cases of childhood leukaemia were diagnosed within the study area compared to 729.09 expected cases, this difference is significant at the 5% significance level (SIR=0.92, CI=[0.85–0.99]). At this level, no evidence was found either of heterogeneity between the four subregions or of a trend of decreasing SIR with distance from the sites based on the same four areas. The latter tests also yielded non significant results based on concentric bands of width 1 km.

The sites of Bruyères, Saclay and Fontenay are located in a very densely populated area and hence account for 471 cases out of the total 670. This inevitably influences the overall tests and calculations in an important way. For this reason, Table 1 also shows the subtotal referring to the 26 sites excluding this group of sites (B/S/F) and the main tests were carried out on this subset of sites as well as on the grand total of 27 sites. There were 199 observed cases around the 26 sites compared to 208.18 expected cases (SIR=0.96, CI=[0.83–1.10]) and, as for all sites, no evidence was found either of heterogeneity between the four subregions or of a trend of decreasing SIR with distance from the sites based on the same four areas.

The numbers of cases according to distance for age groups 0–4, 5–9 and 10–14 years are all lower than expected, although not significantly so (Table 2

**Table 2:** Observed (*O*) and expected (*E*) cases of childhood leukaemia living less than 20 km away from one of the 29 nuclear sites in France (1990–1998), with SIR and their 95% CI, according to age and distance from the nuclear sites

**Age (years)**		**0–5 km**	**5–10 km**	**10–15 km**	**15–20 km**	**Total**
0–4						
	*O*	39	95	114	117	365
	*E*	40.04	92.93	136.33	119.27	388.56
	SIR	0.97	1.02	0.84	0.98	0.94
	95% CI	(0.69–1.33)	(0.83–1.25)	(0.69–1.00)	(0.81–1.18)	(0.85–1.04)
						
5–9						
	*O*	18	38	64	62	182
	*E*	21.68	49.89	71.32	67.64	210.52
	SIR	0.83	0.76	0.90	0.92	0.86
	95% CI	(0.49–1.31)	(0.54–1.05)	(0.69–1.15)	(0.70–1.18)	(0.74–1.00)
						
10–14						
	*O*	8	32	42	41	123
	*E*	13.39	31.48	43.12	42.01	130.00
	SIR	0.60	1.02	0.97	0.98	0.95
	95% CI	(0.26–1.18)	(0.70–1.44)	(0.70–1.32)	(0.70–1.32)	(0.79–1.13)

SIR=standardised incidence ratio; 95% CI=95% confidence interval.

). None of the tests showed any significant association of SIR with distance from the site for any of the age groups, whether considering all sites or the subset of 26 sites.

Significant evidence of heterogeneity between the 27 sites was found using the likelihood ratio test (*P*=0.038). Indeed individual analysis of each site (Table 1) showed two occurrences of a statistically significant excess of cases among children aged 0–14 (Chinon: *O*=14, SIR=2.11, *P*=0.0052; Civaux: *O*=7, SIR=2.37, *P*=0.022) and one statistically significant deficit of cases (Bruyères/Saclay/Fontenay: *O*=471, SIR=0.90, *P*=0.029). These differences do not remain significant after correcting for multiple testing (27 comparisons) by Bonferroni's method. None of the 27 sites present any statistical evidence of a trend in SIR with distance except for Chinon (*P*=0.026) and Creys-Malville (*P*=0.039). This trend is no longer significant after correcting for multiple testing (27 tests) by Bonferroni's method. None of the sites showed any significant heterogeneity between the four subregions except for St Alban (*P*=0.019). No evidence of an interaction between sites and distance was found using a likelihood ratio test based on Poisson regression models.

The 19 EGNS were analysed as a group and according to electric nuclear power and year of start-up. As was the case for the 27 sites' analysis, no evidence was found of heterogeneity according to distance or of a decreasing trend in SIR with distance from the sites, whichever age group was considered.

Table 3

**Table 3:** Observed (*O*), expected (*E*) cases and SIR of childhood leukaemia (0–14 years old) within 20 km of one of the 19 EGNS in France (1990–1998) according to distance from electric nuclear power and start-up year of the EGNS

**Site characteristic**	**Characteristic class values**	**0–5 km**	**5–10 km**	**10–15 km**	**15–20 km**	**Total**
*Electric nuclear power*^a^ *(MWe)*						
1800						
	*O*	1	0	2	2	5
	*E*	0.79	1.96	2.24	5.01	10.00
	SIR	1.27	0.00	0.89	0.4	0.5
						
2600–2800						
	*O*	5	10	7	21	43
	*E*	4.42	6.99	11.09	20.72	43.21
	SIR	1.13	1.43	0.63	1.01	1.00
						
3600						
	*O*	1	16	15	17	49
	*E*	2.63	13.03	11.45	15.44	42.56
	SIR	0.38	1.23	1.31	1.10	1.15
						
5200–5400						
	*O*	1	8	9	10	28
	*E*	*2.80*	*9.15*	*11.43*	*17.87*	41.25
	SIR	0.36	0.87	0.79	0.56	0.68
						
*Start–up year*						
<1980						
	*O*	2	8	9	14	33
	*E*	2.27	6.80	7.38	14.15	30.61
	SIR	0.88	1.18	1.22	0.99	1.08
						
1980–1984						
	*O*	1	11	14	12	38
	*E*	3.62	12.14	14.03	17.70	47.49
	SIR	0.28	0.91	1.00	0.68	0.80
						
1985–1989						
	*O*	5	12	5	12	34
	*E*	3.71	10.27	8.88	21.13	43.99
	SIR	1.35	1.17	0.56	0.57	0.77
						
⩾1990						
	*O*	0	3	5	12	20
	*E*	1.03	1.92	5.92	6.06	14.93
	SIR	0.00	1.56	0.84	1.98	1.34
						
*Total*						
	*O*	8	34	33	50	125
	*E*	10.64	31.13	36.21	59.03	137.01
	SIR	0.75	1.09	0.91	0.85	0.91

EGNS=electricity-generating nuclear sites; SIR=standardised incidence ratio; MWe=mega Watts.

a Electric nuclear power of each EGNS is given by the number of units on site multiplied by the power of each unit (see Table 1 for individual site values).

shows the observed and expected numbers of cases as well as the SIRs according to electric nuclear power, start-up year and distance. No significant variation of SIR according to electric nuclear power was found overall or for each of the four study areas, whether nuclear power was considered quantitatively or qualitatively. We did neither find any evidence of heterogeneity between study areas nor of a decreasing trend in SIR according to distance for any of the classes of nuclear power. Furthermore, there was no evidence of a significant variation of SIR according to year of start-up either overall or for each of the four study areas. No evidence was found of heterogeneity between study areas or of a trend in SIR according to distance from the site for any of the start-up year classes.

## DISCUSSION

This study was designed to detect any evidence of an increased incidence of childhood leukaemia around the 29 French nuclear sites. Overall, the observed number of cases was consistent with the expected number of cases based on national age-specific incidence rates and we did not find any statistical evidence of a decreasing trend in SIR of leukaemia with distance from the sites; this was true for all ages and for each of the three age groups under study.

The use of reliable incidence data rather than mortality data concerning over twice as many nuclear sites considerably increased the power of this study compared to previous French multisite studies (Hill and Laplanche, 1990; Hattchouel *et al*, 1995). The period, age groups and areas under study, namely children aged 0–14 years living within 20 km of one of the 29 nuclear sites during the years 1990 to 1998, were all chosen *a priori* conferring statistical validity and interpretability to our results. Furthermore, the issue of the arbitrary choice of subregions for trend tests is partially solved by the use of Stone's Poisson maximum test. This test has the advantage of being adaptive in the sense that it determines the distance at which the observed effect is maximal while simultaneously adjusting for this selection.

As our study period was placed between two census dates, we were able to use interpolation and thus obtain reliable population estimates. Different methods of interpolation were compared in order to check the reliability of our estimates. These consistently gave extremely close results leading us to believe that our population estimates were largely satisfactory and should not have influenced our results.

On the one hand, none of the sites presented an increased incidence of childhood leukaemia except for Chinon and Civaux and on the other, there was a significant deficit of cases for Bruyères/Saclay/Fontenay. One has to bear in mind that a large number of tests have been carried out. We used Bonferroni's method to correct for multiple testing. The excesses of cases at both Chinon and Civaux and the deficit of cases at Bruyères/Saclay/Fontenay were no longer significant by these standards. Note that the Civaux power plant was in operation for less than 3 years during the study period. Kinlen's population mixing hypothesis (1988, 1995) could possibly explain this excess. However, a much more detailed study, such as that carried out by Boutou *et al* (2002) at La Hague, would be necessary in order to confirm this hypothesis. At Civaux, the SIRs did not decrease significantly with distance from the site. A significant trend in SIRs with distance was found at Chinon (*P*=0.026); however, the trend is no longer significant after Bonferroni's correction. At St Alban, despite an overall deficit of cases (*O*=12, *E*=15.72), there was significant heterogeneity between the four areas under study and the SIR decreased as distance from the site increased. However, this trend was not statistically significant.

The pooled analysis of the 19 EGNS was motivated by their common characteristics. We found no overall excess of cases of childhood leukaemia near these sites during our study period. The tests for detecting a decrease in SIR with increasing distance from the site were not statistically significant. Similar results were obtained when the start-up year of the nuclear sites and their electric nuclear power were taken into account.

Levels of education and unemployment rate in the areas around the sites were found to be similar to the national average, which means social condition is unlikely to have been a confounding factor. Other site-specific factors could have influenced our findings, but using internal references yielded very similar results.

For the pooled analysis of the 27 nuclear sites, the power was excellent for initial SIRs (i.e. in the 0–5 km area) of 2 and 1.5 (96–100% depending on the test and the alternative hypothesis) and fair for an initial SIR of 1.2 (40–95% depending on the alternative hypothesis). All powers were calculated with a 5% probability of type-I error (see Appendix A).

As in most of the current literature on the subject, our study shows no evidence of a generally increased risk of childhood leukaemia within 20 km of the 29 nuclear sites under study during 1990–1998. However, the risk associated with continuous and lasting exposure to very small doses of ionising radiation remains uncertain, and research on radiation-induced risks is still necessary. Systematic surveillance of childhood leukaemia incidence around nuclear installations should continue, along with continuous quantitative measures of dose levels around the sites and radioecological studies based on exact-dose data.

## Appendix A

The statistical power of the global analysis of the 27 nuclear sites to detect a decrease in SIR with increasing distance was examined according to two types of alternative hypotheses, the null hypothesis being that the SIRs (*ρ*_*i*_, *i*=1,…,4) in the four study zones (0–5, 5–10, 10–15 and 15–20 km) equal 1. The first form of alternative hypothesis was that the SIRs decreased linearly with distance according to four initial SIRs, that is, an SIR of 2.0 (respectively 1.5, 1.2 and 1.1) in zone 0–5 km declining linearly with distance to 1.0 in the last zone. Secondly, we considered alternative hypotheses presenting sharp decreases in SIR with distance from the site, where the SIR initially takes value *ρ*_1_ in zone 0–5 km and then drops to 1. The ‘drop’ was chosen to occur 5, 10 and 15 km from the site according to four initial values of the SIR in the first zone. Hence, there were four alternative hypotheses of the linear type and 12 of the sharp decrease type.

The above-mentioned null hypothesis was tested using a linear risk score test with inverse distance as the main parameter and Stone's Poisson maximum test. The statistical power of both these tests for each alternative hypothesis was determined as well as that of the most powerful (or optimal) test (Bithell, 1995). The statistic for this last test depends on the real SIRs and hence on the alternative hypothesis. Under the null hypothesis of a uniform SIR of 1.0 in all four zones around the sites, the null distributions of the three tests were determined from 10 000 simulations based on the expected numbers of cases sampled from the appropriate Poisson distribution. From these null distributions, the 5% critical values of the test statistics were estimated. Owing to its dependence on the real SIRs, the optimal test has a different critical value for each alternative hypothesis. For each of the three tests, 10 000 simulations were carried out under each alternative hypothesis. The proportion of simulated test statistics that exceeded the critical values provided an estimate of the power of each test to reject the null hypothesis at the 5% significance level (Table A1

**Table A1:** Statistical power of the optimal test, the LRS test and the Stone's Poisson maximum test at the 5% significance level according to four values of the SIR

		**Optimal test**			
**H_1_**	***ρ*_1_**	***α*_0_**	**Power**	**LRS test**	**Poisson max. test**
*Linear decrease*					
	1.1	4.79	36.98	32.94	19.60
	1.2	4.95	84.58	77.78	53.48
	1.5	4.71	100.00	100.00	99.89
	2	4.99	100.00	100.00	100.00
					
		***α*_0_**		4.91	3.93
					
*Rapid decrease after 5 km*					
	1.1	4.03	19.00	18.18	19.07
	1.2	5.34	47.22	40.34	47.27
	1.5	4.16	98.40	95.57	98.40
	2	5.49	100.00	100.00	100.00
					
*Rapid decrease after 10 km*					
	1.1	4.52	43.82	33.43	21.13
	1.2	5.05	91.59	78.42	64.60
	1.5	4.57	100.00	99.99	99.98
	2	4.34	100.00	100.00	100.00
					
*Rapid decrease after 15 km*					
	1.1	4.88	71.00	52.59	22.40
	1.2	4.84	99.42	95.41	72.33
	1.5	4.52	100.00	100.00	100.00
	2	4.49	100.00	100.00	100.00
					
		*α***_0_**		4.83	4.10

LRS test=linear risk score test; SIR=standardised incidence ratio. *ρ*_1_ in the 0–5 km zone under two types of alternative hypothesis H_1_: a linear decrease in SIR and three forms of rapid decrease in SIR (after 5, 10 or 15 km); *α*_0_ is the real value of the probability of type-I error (given by simulation). *Note*: These calculations are for the 27 site global analysis.

).

The statistical powers of the linear risk score test and the Poisson maximum test were both close to that of the optimal test under the various alternative hypotheses. The linear risk score test had greater statistical power than Stone's Poisson maximum test for all the patterns of decrease and whatever the initial value of the SIR, the only exception being the assumption of a sharp decrease after 5 km. This is not surprising considering Stone's test was specifically designed for this type of risk pattern. For the pooled analysis of the 27 nuclear installations, the power was excellent for initial SIRs of 2 and 1.5, and fair for an initial SIR of 1.2 (with a 5% probability type-I error). The powers for the detection of an effect when considering the subset of 26 sites or the 19 EGNS alone were slightly lower (data not shown).
